# A novel *SMARCA2-CREM* fusion: expanding the molecular spectrum of intracranial mesenchymal tumors beyond the *FET* genes

**DOI:** 10.1186/s40478-021-01278-4

**Published:** 2021-10-29

**Authors:** Arnault Tauziède-Espariat, Gaëlle Pierron, Delphine Guillemot, Philipp Sievers, Dominique Cazals-Hatem, Thierry Faillot, Alexandre Roux, Joseph Benzakoun, Sophie Bockel, Nicolas Weinbreck, Lauren Hasty, Emmanuèle Lechapt, Fabrice Chrétien, Pascale Varlet

**Affiliations:** 1 Department of Neuropathology, GHU Paris - Psychiatry and Neuroscience, Sainte-Anne Hospital, 1, rue Cabanis, 75014 Paris, France; 2 Institut de Psychiatrie et Neurosciences de Paris (IPNP), UMR S1266, INSERM, IMA-BRAIN, Paris, France; 3 Université de Paris, Paris, France; 4 Paris-Sciences-Lettres, Curie Institute Research Center, INSERMU830, Paris, France; 5 Laboratory of Somatic Genetics, Curie Institute Hospital, Paris, France; 6 Department of Neuropathology, Institute of Pathology, University Hospital Heidelberg, Heidelberg, Germany; 7 Clinical Cooperation Unit Neuropathology, German Cancer Research Center DKFZ, German Consortium for Translational Cancer Research (DKTK), Heidelberg, Germany; 8 Department of Pathology, APHP University Hospital Beaujon, Clichy, France; 9 Department of Neurosurgery, APHP University Hospital Beaujon, Clichy, France; 10 Department of Neurosurgery, GHU Paris-Psychiatrie et Neurosciences, Sainte-Anne Hospital, Paris, France; 11 Department of Radiology, GHU Paris-Psychiatrie et Neurosciences, Sainte-Anne Hospital, Paris, France; 12 Department of Radiation Oncology, Gustave Roussy, Paris-Saclay University, Villejuif, France; 13 Medipath Pathology Lab, Frejus, France

**Keywords:** SMARCA2, CREM, Intracranial mesenchymal tumor

## Abstract

A novel histomolecular tumor of the central nervous system, the “intracranial mesenchymal tumor (IMT), *FET-CREB* fusion-positive” has recently been identified in the literature and will be added to the 2021 World Health Organization Classification of Tumors of the Central Nervous System. However, our latest study using DNA-methylation analyses has revealed that intracranial *FET-CREB* fused tumors do not represent a single molecular tumor entity. Among them, the main subgroup presented classical features of angiomatoid fibrous histiocytoma, having ultrastructural features of arachnoidal cells, for. Another tumor type with clear cell component and histopathological signs of aggressivity clustered in close vicinity with clear cell sarcoma of soft tissue. Herein, we report one case of IMT with a novel *SMARCA2-CREM* fusion which has until now never been described in soft tissue or the central nervous system*.* We compare its clinical, histopathological, immunophenotypic, genetic and epigenetic features with those previously described in IMT, *FET-CREB* fusion-positive. Interestingly, the current case did not cluster with IMT, *FET-CREB* fusion-positive but rather presented histopathological (clear cell morphology with signs of malignancy), clinical (with a dismal course with several recurrences, metastases and finally the patient’s death), genetic (fusion implicating the *CREM* gene), and epigenetic (DNA-methylation profiling) similarities with our previously reported clear cell sarcoma-like tumor of the central nervous system. Our results added data suggesting that different clinical and histomolecular tumor subtypes or grades seem to be included within the terminology “IMT, *FET-CREB* fusion-positive”, and that further series of cases are needed to better characterize them.

## Introduction

FET fusions (encompassing both *EWSR1* and *FUS*) with genes from the CREB (cAMP response element) family (*CREB1*, *CREM* and *ATF1*) are involved in a wide variety of tumors of various locations and prognoses (angiomatoid fibrous histiocytomas, clear cell sarcomas of the soft tissue –CCS-, gastrointestinal neuroectodermal tumors, and primary pulmonary myxoid sarcomas). In the central nervous system (CNS), the recent literature identified a novel histomolecular type, named “intracranial mesenchymal tumor (IMT), *FET-CREB* fusion-positive” which will be added in the new WHO classification [1]. However, a previous study reveals that IMT, *FET-CREB* fused do not represent a single molecular tumor type but different epigenetic subtypes according to DNA-methylation analyses [2]. The main methylation class is characterized by an extra-axial neoplasm in adults, with syncytial or spindle cells, in a mucin or collagenous-rich stroma. The immunoprofile is variable but EMA and desmin are frequently expressed. This main cluster due to arachnoidal characteristics, justifies the terminology “intracranial mesenchymal tumors” (IMT) [2–23]. However, a minority of cases clustered near clear cell sarcoma or extra-CNS angiomatoid fibrous histiocytomas. Herein, we report one case of IMT with a novel *SMARCA2-CREM* fusion*.* We compare its clinical, histopathological, immunophenotypic, genetic and epigenetic features with those previously described in IMT, *FET-CREB* fusion-positive.

### Case presentation

**Fig. 1:**
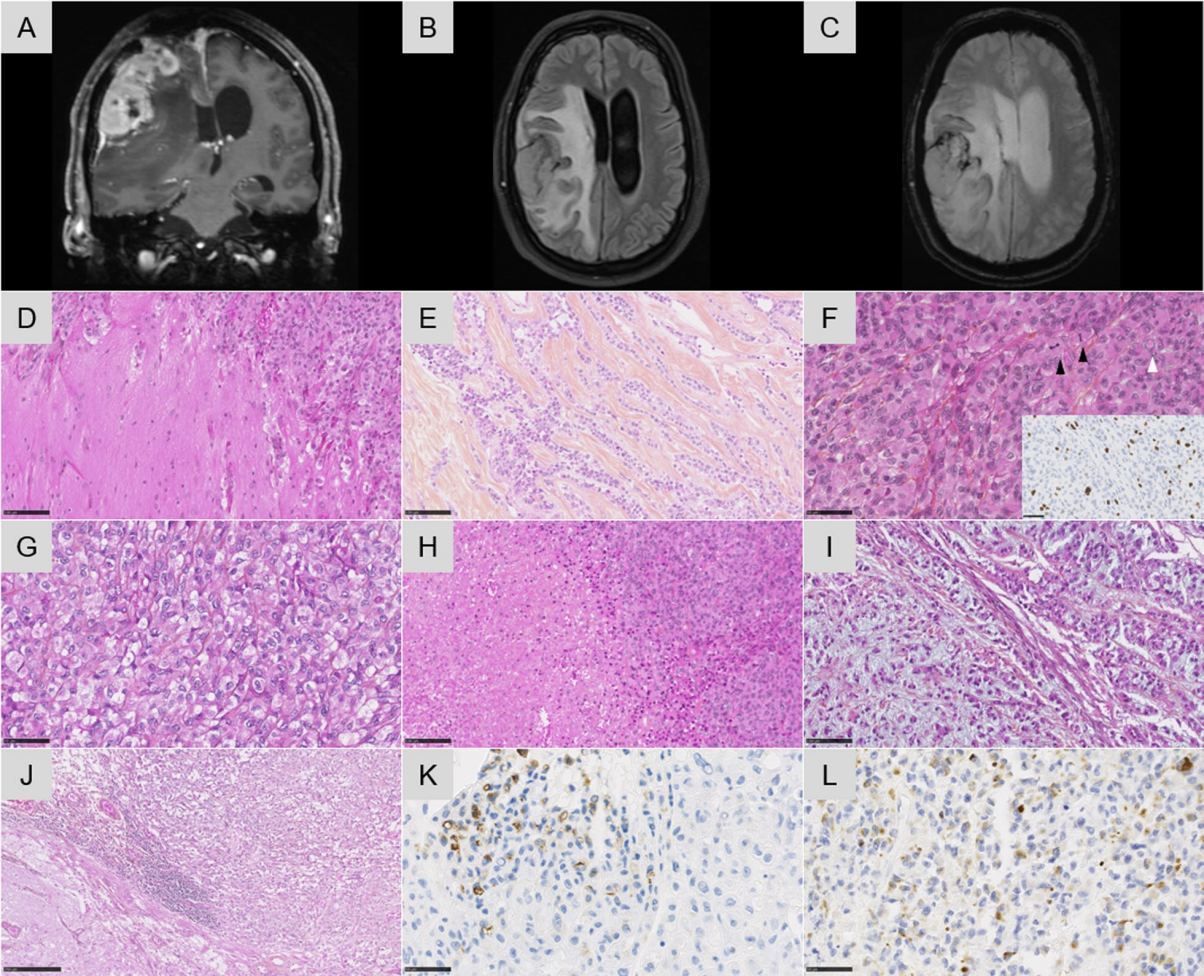
Radiological and histopathological features of the recurrent tumor **A** Coronal T1-weighted magnetic resonance imaging sequence after contrast injection showing an enhancing extra-axial right parietal mass with necrotic component and dural tail. **B** Axial Fluid attenuated inversion recovery (FLAIR) sequence showing a perilesional edema. **C** Axial T2*-weighted sequence showing anterior hemorrhagic modifications (low-intensity areas). **D** Infiltration of the brain parenchyma by tumor cells (HPS, magnification × 400). **E** Collagen deposits in the tumor (HPS, magnification × 400). **F** Proliferation composed of epithelioid cells with intranuclear pseudoinclusions (white arrowhead) and several mitoses (black arrowhead) and elevated MIB-1 labeling index (HPS, magnification × 400, and insert MIB-1 magnification × 400). **G** Clear cell component (HPS, magnification × 400). **H** Necrosis (HPS, magnification × 400). (**I**) Chordoid inflexion (HPS, magnification × 400). **J** Peritumoral inflammatory infiltrates (HPS, magnification × 100). **K** EMA immunoexpression by tumor cells (magnification × 400). **L** Desmin immunoexpression by tumor cells (magnification × 400). Black scale bars represent 50 µm for figures D-I and K-L; and 250 µm for figure J. HPS: Hematoxylin Phloxin Saffron

**Fig. 2:**
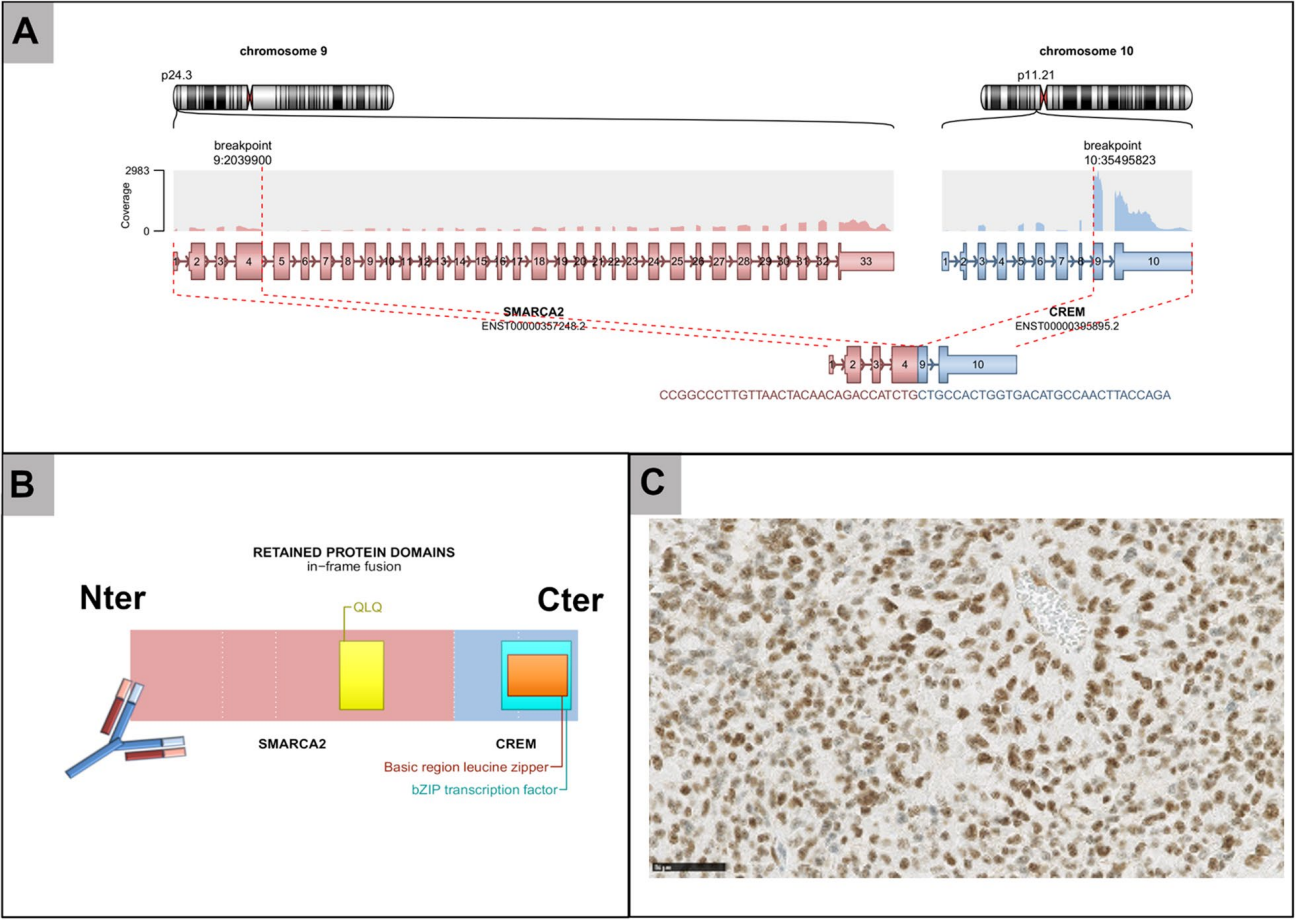
Genetic features. **A** RNAseq analysis highlights a fusion between *SMARCA2* (pink) and *CREM* (blue) genes, respectively located on chr9p24.3 and chr10p11.21. As the breakpoints are intra exonic (in exon 4 for *SMARCA2*, and exon 9 for *CREM*), the fusion point can easily been detected by split and span reads encompassing the rearrangement with a good coverage. **B** Chimeric protein between SMARCA2 and CREM with retained protein domain of SMARCA2 detected by the immunohistochemical antibody. **C** Immunopositivity for SMARCA2 in the current tumor (magnification, 400x)

**Fig. 3:**
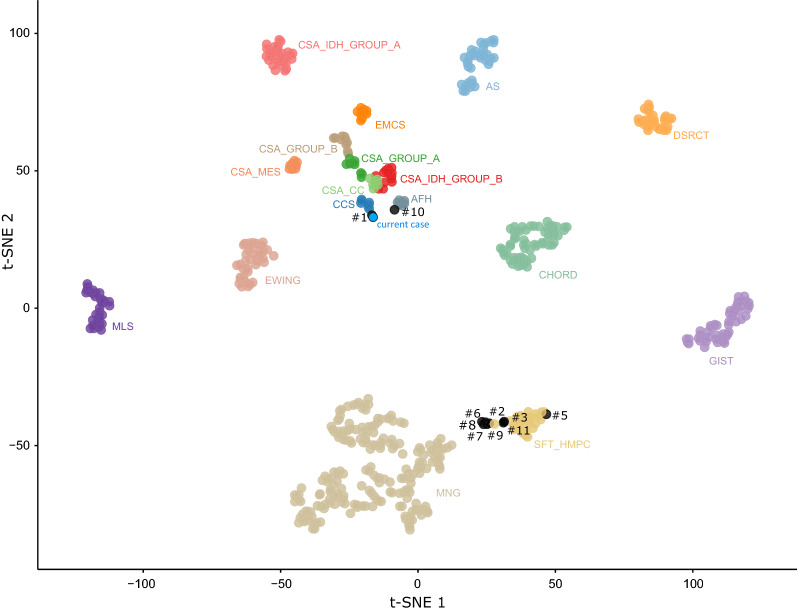
Methylation-based t-SNE distribution. Reference DNA methylation classes: angiomatoid fibrous histiocytoma (AFH), angiosarcoma (AS), clear cell sarcoma of soft parts (CCS), chordoma (CHORD), clear cell chondrosarcoma (CSA_CC), chondrosarcoma group A (CSA _Group_A), chondrosarcoma group B (CSA_Group_B), chondrosarcoma IDH mutant group A (CSA_IDH_Group_A), chondrosarcoma IDH mutant group B (CSA_IDH_Group_B), chondrosarcoma mesenchymal (CSA_MES), desmoplastic small round cell tumour (DSRCT), extraskeletal myxoid chondrosarcoma (EMCS), ewing sarcoma (EWING), gastrointestinal stromal tumour (GIST), myxoid liposarcoma (MLS), meningioma (MNG), solitary fibrous tumor / hemangiopericytoma (SFT_HMPC). Previous reported cases [2] are designated in black, the current case is designated in blue

A 41-year-old man presented with an extra-axial heterogeneous mass in the right parietal region with necrotic and hemorrhagic components (Fig. 1A–C). The patient has a history of chordoid meningioma in the same area, and has been treated for 15 years by several resections and radiation therapy due to local recurrences (despite an initial gross total resection). This novel local progression was surgically excised. Microscopically, the tumor showed a multinodular, densely cellular proliferation, infiltrating the brain parenchyma (Fig. 1D). The tumor presented thick fibrous bands (Fig. 1E) and the tumor cells had an epithelioid morphology with prominent nucleoli, intranuclear inclusions but no psammoma bodies or whorls (Fig. 1F). The cytoplasm of the tumor cells was abundant, eosinophilic (Fig. 1F) or clear (Fig. 1G). Signs of malignancy were obvious high mitotic index with 21 mitoses per 1.6 mm^2^, and elevated MIB-1 labeling index (Fig. 1F insert) and necrosis (Fig. 1H). In some areas, the tumor cells presented rhabdoid or chordoid features (Fig. 1I). Whereas the tumor exhibited scattered inflammatory infiltrates, the adjacent brain parenchyma showed several inflammatory foci of lymphoplasmocytic cells (Fig. 1J). The tumor cells were immunonegative for CKAE1/AE3, SSTR2A, CD99, NUT, Olig2, GFAP, SOX10, PS100, HMB45, chromogranin A, synaptophysin, neurofilaments, MUC4 and smooth muscle actin. They only focally expressed EMA and desmin (Fig. 1K–L), and more diffusely CD68. The expression of BRG1, INI1 and H3K27me3 was retained. The current tumor was compared with previous surgical samples and was histopathologically similar (high mitotic index with 12 mitoses per 1.6 mm^2^, and elevated MIB-1 labeling index but without necrosis initially). Moreover, initial and recent imaging of the whole body did not show other tumor locations. FISH analysis of the *EWSR1* gene failed to reveal a rearrangement and RNA sequencing evidenced a *SMARCA2-CREM* gene fusion (Fig. 2A–B). This fusion was also found in the primary tumor by RNA sequencing analysis. An additional immunostaining for SMARCA2 was secondarily performed but revealed a retained expression of the protein (Fig. 2C) so a DNA-methylation analysis was conducted. The tumor was not classifiable using the Heidelberg Brain Tumor or Sarcoma Classifiers (v11b4/v12.2). Next, a t-Distributed Stochastic Neighbor Embedding (t-SNE) analysis was performed in comparison with the genome-wide DNA methylation profiles of the sarcoma reference cohort as well as a more focused analysis with selected reference groups including in particular, angiomatoid fibrous histiocytomas and CCS of soft tissue, meningiomas, and the previously reported *FET:CREB* intracranial tumors cohort [2]. By using unsupervised t-SNE and by RNA seq clustering, the tumor was in close vicinity with the cluster of CCS and case 1 of our previous study (Fig. 3) [2]. A few months later, the patient presented secondary locations in the lungs and died rapidly of the disease, 184 months after the initial diagnosis.

## Discussion and conclusions

IMT are characterized by recurrent *FET-CREB* translocations, always involving the *FET* genes to date, mainly *EWSR1* and exceptionally *FUS* [3–9, 11–23]. *SMARCA2-CREM* fusion has not been previously reported in CNS or in soft tissue. The *SMARCA2* gene, located at 9p24.3, belongs to the SWI/SNF family, responsible for chromatin remodeling repair, and has been implicated in a wide spectrum of tumors, such as carcinomas (*SMARCA2* mutations) and sarcomas (concomitant loss of SMARCA4 and SMARCA2 expressions) [24, 25]. Only one case of extraskeletal myxoid chondrosarcoma of the foot was described with a *SMARCA2-NR4A3* fusion (with the same breakpoint at *SMARCA2* gene) [26]. As was the case with our CNS tumor, this reported case presented a retained immunoexpression of SMARCA2 [26]. As the SMARCA2 antibody is designed to bind to the Nter domain of the native protein, this epitope is maintained in the resulting chimeric fusion protein and detected by immunohistochemistry (Fig. 2). Interestingly, as in our case, the common fusion gene partner (*EWSR1*) implicated in extraskeletal myxoid chondrosarcomas is replaced by the *SMARCA2* gene, suggesting a similar molecular tumorigenesis. Previously, a wide spectrum of histopathology has been reported in *FET-CREB* fused tumors of the CNS, including chordoid morphology [20], suggesting that specific differences in the fusion protein or specific cell context features may be critical determinants of tumor morphology [2]. However, a recent study has identified that despite this morphological and epigenetic heterogeneity, all CNS tumors presented ultrastructural homologies suggesting an arachnoidal cell origin [2]. The current tumor shares some features of IMT, *FET-CREB* fusion-positive but did not cluster with them when studied using DNA-methylation analysis. Moreover, it presents similar clear cell morphology, radiological features, histopathological signs of malignancy, pejorative outcome, *CREM* gene fusions, and epigenetic features to one reported CCS-like tumor of the CNS [2].

In conclusion, we expanded the IMT, *FET-CREB* fused genetic spectrum with one novel fusion that does not involve the *EWSR1* gene. This current case constitutes the second CCS-like tumor having a worse prognosis than IMT, *FET-CREB* fusion-positive. Further studies are needed to characterize in detail this rare type of tumor and to validate the existence of different epigenetic tumor subtypes.
